# Remarks on Myocarditis Associated with COVID-19 Infection and Myocarditis Following mRNA COVID-19 Vaccination

**DOI:** 10.3390/jcm11216511

**Published:** 2022-11-02

**Authors:** Andrea Frustaci

**Affiliations:** Cellular and Molecular Cardiology Lab, IRCCS L. Spallanzani, 00149 Rome, Italy; biocard@inmi.it

COVID-19 virus infection is responsible for one of the worst reported pandemics as of August 2022, of 585 million human infections and 6.4 million deaths [1] in the world. While the modalities of disease transmission through the respiratory system, as well as the role of spike protein and ACE receptors for viral uptake and cell internalization, are well defined, individual susceptibility to viral infection is still unclear. Indeed, it has been observed that even in the context of the same family, some patients have a mild or a moderate form of the disease and recover spontaneously, while others have a severe manifestation and die. A recent report demonstrates that HLA variation affects the cellular immune response to peptides from human-infecting coronaviruses with HLA-B*46:01, causing increased HLA-B*15:01 and reduced vulnerability to viral infection [2].

It is likely that a blood screening of HLA system could predict individual susceptibility to COVID-19 infection before its exposure.

Regarding the viral involvement of cardiovascular system, cardiac injury has been observed in approximately 30% of COVID-19 infections and has been associated with an adverse prognosis. The clinical presentation of cardiac involvement has been mostly represented by COVID-19-related myo-pericarditis.

Post-mortem studies report myopericarditis in approximately 30% of cases with rare identification by the polymerase chain reaction of COVID-19 genomes [3]. The mechanisms involved in cardiac damage are mostly considered immune-mediated with deposition in the myocardium and cardiac vessels of immune complexes as a consequence of the cytokine storm. The type of damage is a lymphocytic inflammation affecting pericardium and practically all components of the myocardium. Indeed, in addition to the necrosis of cardiomyocytes, the vasculitis of intramural vessels, the infiltration of conduction tissue, and the necrotic damage of subepicardial ganglia are recognized [4], the involvement of these two last targets may explain the relief of electrical instability and the occurrence in these patients of sudden cardiac death.

On therapeutic ground, several drugs, including antiviral agents such as Redemsivir and Lopinavir + Ri- tonavir, antibiotics such as azithromycin, and anti-inflammatory agents such as hydroxychlorochine, have been applied with doubtful effects on the clinical outcome. Steroids, particularly in compromised patients, have been found to diminish myocardial oedema, improve systolic function, and reduce mortality rate by 35% [5], hinting that immune-mediated damage is a consistent operative mechanism that can be pharmacologically modulated, improving patients’ prognosis.

Myocarditis after the BNT162b2 mRNA anti-COVID-19 vaccine has been reported in around 1 on 100,000, and is higher in males than in females with top incidence in young males, aged 16–19 years [6]. The incidence of post-vaccination myocarditis appears mildly more pronounced following mRNA-1273 (Moderna) administration [6]. Clinically, it manifests a few days after vaccine administration and is considered unlikely connectable to vaccination after 4 weeks.

Diagnosis is usually obtained through clinical considerations and non-invasive investigations, including ECG, echocardiogram, troponin I determination, and cardiac magnetic resonance imaging. Endomyocardial biopsy has been undertaken in a very limited number of cases and this limits the available knowledge on the pathway.

Interestingly, infection with SARS-CoV2 increases the risk of myocarditis by 16-fold from 9 per 100,000 to 150 cases per 100,000 [7], and seems to be more severe.

On the other hand, myocarditis following COVID-19 vaccination is reported as benign in 95% of cases with spontaneous resolution and very occasional fulminant forms and death [8].

The mechanism involved in post-vaccination myocarditis is still unclear, as are the methods used to recognize people potentially susceptible to this adverse reaction. Endomyocardial biopsy [9] and post-mortem studies [10] suggest hypersensitivity to spike protein to be a likely possibility. Indeed, the abundance of eosinophils among myocardial inflammatory infiltrates as well as the degranulation of the crystalloids of eosinophils support this hypothesis. On this basis, patients with allergic diseases appear to be at a higher risk of developing post-vaccine myocarditis, while the elevation of plasma cationic protein would become a diagnostic biomarker of this complication.

Although the above-mentioned speculations need a final general consensus, the administration of moderate doses of steroids for 2–4 weeks appears highly effective in the termination of myocardial inflammation.
